# Radical Operation for Varicose Veins

**Published:** 1897-05

**Authors:** 


### RADICAL OPERATION FOR VARICOSE VEINS.

O'Conor (*Annals of Surgery*, April, 1897, p. 526) describes briefly the mode of procedure that he has followed during a year in the radical treatment of varicoseveins. The limb, having been shaved and disinfected from Poupart's ligament to the ankle, a two-inch incision is made over the saphenous opening and the internal saphenous trunk is doubly ligated and divided; if no varicosity is present above the knee, the wound is closed and dressed at once with iodoform gauze. If the femoral portion is affected, after ligation at the saphenous opening the vein is dissected up and its tributaries seized with pressure forceps and ligated. In nearly all cases, if varices are present above the knee, there are also some below. Consequently the incision is prolonged downward directly over the vessel until the lowest limit of the disease is reached; the vein is then tied and divided below. In some cases an incision of eighteen or twenty inches is necessary. If the disease does not extend above the knee, after occluding the saphenous trunk in the manner described an incision is made over the affected portion, a ligature is applied above and below and the whole mass is removed by dissection. This maneuver, it is said, may be carried out with surprising ease and rapidity. All branches are caught up with pressure forceps, and when the main channel is removed they are ligated. As frequently the external saphenous vein is also affected, its varicose portion is dealt with in a similar manner. While, to one unaccustomed to an incision of eighteen or twenty inches, this plan may seem formidable, it is pointed out that if the vessel is ligated above and below the varicose area there is not the slightest danger of embolism or pyemia, and the hemorrhage is insignificant. The method has also been employed in the removal of large thrombosed veins occurring in the first few months of pregnancy. Under these circumstances, above all others, it is particularly necessary, before manipulating the diseased portion, to occlude the main vein well above the seat of the disease, so that if thrombi are dislodged they can not pass into the general circulation. The time occupied in this procedure need not be greater than is required in other comparable operations. The wounds made heal just as kindly as do the short ones, and with ordinary surgical cleanliness there is nothing to be feared. The insertion of a strand of iodoform gauze as a drain to every four inches of wound is a useful precaution, for it does away with the risk of any collection of blood. No bad results were observed to follow the employment of this method, and the patients treated are grateful for the results.

